# Twenty-Year Trends of Low Birth Weight in Japan: A Joinpoint Regression Analysis of Data From 2000 to 2019

**DOI:** 10.3389/frph.2021.772575

**Published:** 2021-12-15

**Authors:** Tomosa Mine, Satoshi Tsuboi, Fujiko Fukushima

**Affiliations:** ^1^Department of the Scientific Study of Children, Shokei Gakuin University, Natori, Japan; ^2^Department of Hygiene and Preventive Medicine, Fukushima Medical University, Fukushima, Japan; ^3^Department of Nursing, Toho University, Ota-Ku, Japan

**Keywords:** low birth weight, joinpoint analysis, Japan, long-term trend data, singleton births, full term birth

## Introduction

Low birth weight (LBW), defined as a birth weight <2,500 g, is a major public health concern worldwide. Globally, in 2015, 20.5 million infants with LBW were born, accounting for 14.6% of all births, according to data compiled by United Nations International Children's Emergency Fund (1). According to the World Health Organization, the proportion of infants with LBW offers an indicator of multifaceted public health problems, including long-term maternal malnutrition, ill-health, and poor healthcare in pregnancy (2). In addition, the Developmental Origins of Health and Disease theory states that in addition to neonatal mortality and morbidity, LBW is associated with long-term health conditions such as cardiovascular disease, type II diabetes, and hypertension (3). To manage maternal and child health efficiently at the population level, careful observation of LBW is necessary.

In 2019, 9.4% of babies born in Japan were LBW (4), higher than the 2018 average among Organisation for Economic Co-operation and Development (OECD) countries of 6.6% (5). For example, 2019 prevalences were 6.8% in the United Kingdom, 8.3% in the United States, and 6.6% in South Korea (5). Japan has one of the highest prevalences of LBW, accounting for about 9–10% of births in recent decade. Many developed countries have shown increases in the prevalence of LBW since 2000, most particularly Southern European countries such as Greece 9.9% (2019), Portugal 8.9% (2019), and Spain 7.5% (2019). However, in Japan, the LBW situation differs from that observed in such countries. Population-based Japanese vital statistics data show that the rate of neonates with LBW has almost doubled from 5.1% in 1975 to 9.5% in 2005 (4). Over the past three decades, the rate of increase in LBW has appeared more rapid in Japan than in many developed countries.

Although early gestational age (6) and multiple births (7) are known risk factors for LBW, previous studies and reports on LBW in Japan have shown trends in the prevalence of all LBW without accounting for the effects of gestational age or multiple births (8, 9). However, half of Japanese cases of LBW fall into the category of singleton, full-term births (8). Little scientific evidence has thus been accumulated regarding trends in Japanese LBW among full-term births. LBW is considered a persistent and severe issue over the long term in Japan. Accordingly, observation of the trends and discussion of potential approaches to preventing LBW in Japanese infants after excluding preterm and multiple births is meaningful.

The present study aimed to observe the 20 year trend between 2000 and 2019 in the prevalence of LBW in singleton, full-term births in Japan and conduct joinpoint regression to analyze trends in detail. It is hoped that this study will provide new insights that will prove useful for maternal and child health.

## Methods

### Study Design

This study was a retrospective, nationwide, population-based, observational study of vital statistics published by the Japanese government (4). Secondary analysis was performed based on governmental statistical data from 2000 to 2019, to observe long-term trends in the prevalence of LBW.

### Setting and Data

The number of births and LBW newborns by gestational age and sex were obtained from annual vital statistics population data collected by the Ministry of Health, Labor and Welfare based on birth certificates. The dataset used in this study showed only the annual number of births according to gestational age, sex, and birth weight, and did not include information on individuals. In Japan, birth certificates are issued by obstetricians and midwives in hospitals and clinics at the time of delivery, and these data are reported to the mayor of the municipality. The birth certificate contains information such as sex, birth weight, gestational age multiple pregnancies, ages of the father and mother, and place of birth. These data are electronically registered and systematized by the municipality as vital statistics data.

### Outcome Variables

Outcome variables for this study were the prevalence of LBW in singleton, full-term births in Japan. We calculated the prevalence of presented variables as the percentage (i.e., number of live-born babies with birth weight <2,500 g in singleton, full-term birth/total number of live-born babies in singleton, full-term births) ^*^ 100.

We limited our analysis to singleton, full-term births to avoid bias from multiple births and gestational age as risk factors for LBW. Based on birth weight and gestational age, we defined LBW in full-term birth as a birth weight of <2,500 g at a gestational age of ≥37 weeks.

### Statistical Analyses

Trends in the prevalence of LBW from 2000 to 2019 were analyzed using joinpoint regression analysis. This method connects several different line segments, thus allowing for succinct characterization of changes in a trend over time (10). The Joinpoint Regression Program version 4.6.0.0 software was provided by the Surveillance Research Program of the National Cancer Institute. Tests for fit in a maximum of four joinpoints (11). In this study, joinpoint regression analysis was used to identify *years* (as the independent variable) in which significant changes in prevalence rate occurred over the study period, and amount of change as the annual percentage change in rate per year. We allowed up to four joinpoints and identified any difference from no change in each segment, with values of *p* < 0.05 considered statistically significant. Stata for Mac version 15 (Stata Corp, College Station, Texas, USA) was used for all other statistical analyses.

### Ethical Aspects

The Ethics Committee for Human Subjects Research at Shokei Gakuin University waived the requirement for ethical approval based on the use of published, public-domain data.

## Results

**Figure 1:**
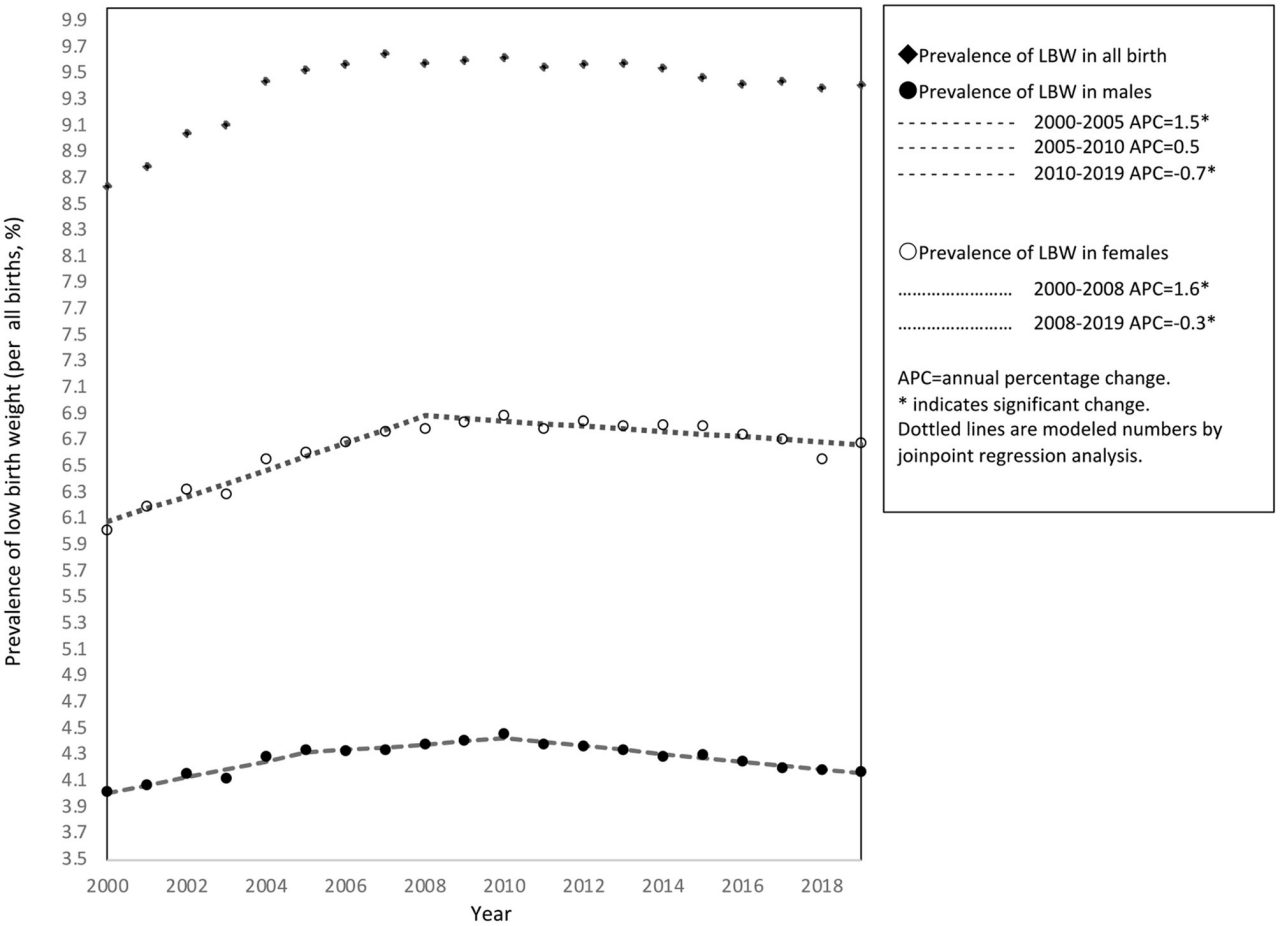
Trends in the prevalence of low birth weight in singleton, full-term births by sex in Japan, 2000–2019.

A total of 21,061,052 births were recorded during the study period, of which 1,976,911 (9.39%) were LBW. Among these, 1,066,959 (53.97%) were singleton, full-term births. Table 1 shows the crude prevalence of LBW in singleton, full-term births according to sex. During 2000–2019, the prevalence of LBW in singleton, full-term births increased from 4.02% (2000) to 4.46% (2010) in males, and from 6.02% (2000) to 6.89% (2010) in females. During this study period, the prevalence of LBW was highest among both males and females in 2010 (Figure 1).

**Table 1:** Birth rate data for all births and low birth weight (LBW) in singleton births in Japan from 2000 to 2019.

**Total (*****n*** **= 21,061,052)**				**Singleton**					
				**All (*****n*** **= 20,630,291)**			**Full-term (*****n*** **= 19,670,578)**		
**Year**	**Births**	**LBW**	**(%)**	**Births**	**LBW**	**(%)**	**Births**	**LBW**	**(%)**
2000	1,190,547	102,888	8.64	1,166,926	86,522	7.41	1,114,345	55,699	5.08
2001	1,170,662	102,881	8.79	1,147,496	86,598	7.55	1,096,877	56,068	5.20
2002	1,153,855	104,314	9.04	1,129,250	86,934	7.70	1,079,223	56,329	5.37
2003	1,123,610	102,320	9.11	1,098,800	84,674	7.71	1,049,408	54,390	5.25
2004	1,110,721	104,832	9.44	1,085,564	86,671	7.98	1,036,010	55,958	5.65
2005	1,062,530	101,272	9.53	1,038,400	83,694	8.06	990,976	54,021	5.30
2006	1,092,674	104,559	9.57	1,068,135	86,649	8.11	1,019,105	55,901	5.50
2007	1,089,818	105,164	9.65	1,065,737	87,606	8.22	1,015,901	56,148	5.51
2008	1,091,156	104,479	9.58	1,068,797	88,140	8.25	1,018,535	56,646	5.67
2009	1,070,035	102,671	9.60	1,049,141	87,281	8.32	999,685	55,953	5.59
2010	1,071,304	103,049	9.62	1,051,103	88,151	8.39	1,001,036	56,545	5.76
2011	1,050,806	100,378	9.55	1,031,187	85,912	8.33	981,685	54,603	5.64
2012	1,037,231	99,311	9.57	1,017,164	84,688	8.33	968,446	54,105	5.63
2013	1,029,816	98,624	9.58	1,009,810	83,997	8.32	961,204	53,316	5.69
2014	1,003,539	95,768	9.54	984,051	81,783	8.31	937,116	51,816	5.51
2015	1,005,721	95,208	9.47	986,253	81,352	8.25	939,966	52,022	5.70
2016	977,242	92,102	9.42	957,874	78,400	8.18	912,846	49,974	5.66
2017	946,146	89,360	9.44	927,105	75,723	8.17	883,018	47,943	5.59
2018	918,400	86,269	9.39	899,661	72,850	8.10	857,340	45,887	5.68
2019	865,239	81,462	9.41	847,837	69,040	8.14	807,856	43,635	5.40

Figure 1 also shows the results of joinpoint regression analysis. Two joinpoints were identified in males, in 2005 and 2008; and one joinpoint in females, in 2008. The prevalence of LBW in males increased by 1.5% (95% confidence interval [CI]: 1.0% to 1.9%) per year until 2005, then decreased by 0.7% (95%CI: −0.5% to −0.9%) per year between 2010 and 2019. In the 2005–2010 period, the gradual increase in prevalence among males was not significant (95%CI: −0.1% to 1.2%). Among females, the prevalence increased by 1.6% (95%CI: 1.3% to 1.9%) per year until 2008, then decreased by 0.3% (95%CI: −0.5% to −0.1%) between 2008 and 2019.

## Discussion

To the best of our knowledge, the current study is the first to quantify upward and downward trends in the prevalence of singleton, full-term LBW in Japan. Joinpoint regression analysis was performed in a large, long-term, national dataset covering almost all births for the period from 2000 to 2019. The first 10 years showed an upward trend, and the second 10 years showed a downward trend, with peak prevalence occurring in 2010 for both males (4.46%) and females (6.89%). Joinpoint analysis also revealed the statistical significance of change points for the start points of decreases in the prevalence of LBW in singleton, full-term births (2010 in males, 2008 in females). Takemoto et al. studied singleton births between 1979 and 2010 using vital statistics data and reported a greater increase in the prevalence of LBW among full-term births in the studied period, from 2.7% in 1979 to 5.3% in 2010 (8). Most research to date has focused on the increasing prevalence of LBW (12, 13). However, the results of our study showed that within the population of singleton, full-term births, the prevalence of LBW began to decrease around 2010. We thus identified the years in which significant changes occurred in trends for the prevalence of LBW in singleton, full-term births. This provides a clearer overall picture that should help identify areas for research in future work.

Several factor may have contributed to the change in Japanese LBW trends from upward to downward at around 2010. Taken overall, we consider that environmental (including potential consequences of treatment and public health strategies), dietary, and socioeconomic factors are likely to have affected these trends. An ecological study by Erasan et al. reported that factors influencing increasing LBW prevalence in OECD countries between 2000 and 2015 were healthcare coverage, public health system coverage, such as hospitals per million inhabitants, and ratios of healthcare workers, midwives per 1,000 inhabitants (14). Erasan et al. also pointed out an association between some economic and health care system organization and funding and the LBW rate. The discussion of LBW needs to include not only maternal factors, but also environmental factors. Among these, further in-depth analysis is necessary to elucidate the impact of changes to guidelines in 2006 regarding gestational weight gain for pregnant women on both LBW trends and the prevalence of underweight women of reproductive age (12, 15). In addition, careful monitoring of trends in LBW among women with preterm and multiple births is needed to clarify overall Japanese trends in LBW according to strata for gestational age and multiple births. Differences in these trends between singleton, multiple, preterm, and full-term births will provide essential insights to inform public health strategies and thus enable more effective prevention of LBW.

The present study showed several limitations that need to be considered when interpreting the results. First, as we used governmental reports that provided only annual aggregated statistics for sex, gestational age, and birth weight, there was no source of individual-level data. In addition, in contrast to similar previous studies, we could report only crude data regarding the prevalence of LBW, without adjusting for maternal age (16), pre-pregnancy body mass index (17), smoking rate during pregnancy (18), and socioeconomic factors (19), as we were lacking this information.

## Conclusion

This study used joinpoint analysis to demonstrated that the trend in the prevalence of Japanese LBW among the population limited to singleton, full-term births has been decreasing since around 2010. This findings runs counter to existing evidence based on all births, including preterm and multiple births, that the prevalence of LBW has been consistently increasing. Further studies are needed to confirm factors affecting changing trends in the prevalence of LBW.

## Data Availability Statement

Publicly available datasets were analyzed in this study. This data can be found here: https://figshare.com/s/444100c4a0f23b01aac3.

## Author Contributions

Material preparation, data collection, and analysis were performed by TM. The first draft of the manuscript was written by TM. All authors contributed to the study conception and design, commented on previous versions of the manuscript, and have read and approved the final version of the manuscript.

## Funding

This work was supported by a JSPS KAKENHI grant (No. 19K19440) from the Ministry of Education, Culture, Sports, Science and Technology, Japan.

## Conflict of Interest

The authors declare that the research was conducted in the absence of any commercial or financial relationships that could be construed as a potential conflict of interest.

## Publisher's Note

All claims expressed in this article are solely those of the authors and do not necessarily represent those of their affiliated organizations, or those of the publisher, the editors and the reviewers. Any product that may be evaluated in this article, or claim that may be made by its manufacturer, is not guaranteed or endorsed by the publisher.
